# Antibodies against EGF-like domains in *Ixodes scapularis* BM86 orthologs impact tick feeding and survival of *Borrelia burgdorferi*

**DOI:** 10.1038/s41598-021-85624-5

**Published:** 2021-03-17

**Authors:** Juraj Koči, Sandhya Bista, Payal Chirania, Xiuli Yang, Chrysoula Kitsou, Vipin Singh Rana, Ozlem Buyuktanir Yas, Daniel E. Sonenshine, Utpal Pal

**Affiliations:** 1 Department of Veterinary Medicine, University of Maryland, College Park, MD 20742 USA; 2 Institute of Zoology, Slovak Academy of Sciences, Dúbravská cesta 9, 84506 Bratislava, Slovakia; 3 Institute of Virology, Biomedical Research Center, Slovak Academy of Sciences, Dúbravská cesta 9, 84505 Bratislava, Slovakia; 4 Department of Microbiology and Clinical Microbiology, Faculty of Medicine, Istinye University, Zeytinburnu, İstanbul, 34010 Turkey; 5 Department of Biological Sciences, Old Dominion University, Norfolk, VA 23529 USA; 6 Virginia-Maryland Regional College of Veterinary Medicine, College Park, MD USA

**Keywords:** Proteins, Diseases

## Abstract

*Ixodes scapularis* ticks transmit multiple pathogens, including *Borrelia burgdorferi* sensu stricto, and encode many proteins harboring epidermal growth factor (EGF)-like domains. We show that *I. scapularis* produces multiple orthologs for Bm86, a widely studied tick gut protein considered as a target of an anti-tick vaccine, herein termed as Is86. We show that Is86 antigens feature at least three identifiable regions harboring EGF-like domains (termed as EGF-1, EGF-2, and EGF-3) and are differentially upregulated during *B. burgdorferi* infection. Although the RNA interference-mediated knockdown of *Is86* genes did not show any influences on tick engorgement or *B. burgdorferi* sensu stricto persistence, the immunization of murine hosts with specific recombinant EGF antigens marginally reduced spirochete loads in the skin, in addition to affecting tick blood meal engorgement and molting. However, given the borderline impact of EGF immunization on tick engorgement and pathogen survival in the vector, it is unlikely that these antigens, at least in their current forms, could be developed as potential vaccines. Further investigations of the biological significance of Is86 (and other tick antigens) would enrich our knowledge of the intricate biology of ticks, including their interactions with resident pathogens, and contribute to the development of anti-tick measures to combat tick-borne illnesses.

## Introduction

Lyme disease, a prevalent arthropod-borne disease in North America and Europe, is caused by a bacterial pathogen, *Borrelia burgdorferi*, and is transmitted by infected *Ixodes scapularis* and other closely-related ticks via feeding on animals, including humans^1^. Once transmitted to hosts, spirochetes can colonize a variety of organs, causing Lyme arthritis, carditis, and an array of neurological syndromes^2^. Antibiotic therapy resolves clinical symptoms, in most cases, during the early stages of infection. However, persistent or relapsing symptoms (e.g., fatigue, musculoskeletal pain, and cognitive difficulties) can later develop in a subset of patients; these symptoms are collectively referred to as chronic Lyme disease, otherwise known as post-treatment Lyme disease syndrome (PTLDS)^3^. The underlying mechanisms, pathogenesis, and treatment of PTLDS remain unknown^4,5^. Therefore, it is of great importance to develop a vaccine that will prevent the incidence of serious tick-borne infections such as Lyme borreliosis. Most research efforts focus on the identification of either *B. burgdorferi* antigens or tick proteins that are required for the survival of spirochetes within ticks, in an attempt to interfere with pathogen transmission from ticks or infectivity in the hosts, thereby preventing Lyme disease^6^. In fact, a human vaccine based on a recombinant form of a *B. burgdorferi* outer surface protein, OspA^7^, was developed and approved in 1998 by the Federal Drug Administration, but it was later withdrawn because of sales issues and patient-related complications. Other strategies, such as controlling tick infestations, might serve as alternative preventive methods to reduce the incidence of Lyme disease.

*Ixodes* ticks can transmit pathogens to humans and cause a range of serious diseases^8^. Given the lack of effective vaccines, tick-borne diseases continue to spread, impacting human and animal health on a global scale. A traditional way to combat tick-borne diseases is to control the tick population using acaricides, which are becoming increasingly less effective due to the emergence of resistant tick strains. Other preventive measures, including tick avoidance, protective clothing, and tick repellents, are only 20–40% effective^9^. Hence, current efforts have been geared towards the development of highly efficacious anti-tick vaccines to control tick infestations^10–12^. Generally, anti-tick vaccine development is based on tick antigens that are accessible to host-derived antibodies, such as surface-exposed gut antigens. A ‘concealed’ midgut protein from *Rhipicephalus* (formerly *Boophilus*) *microplus* cattle ticks, termed as Bm86, is expressed specifically in the tick midgut with upregulation during feeding^13^; it has been used as a commercially available anti-tick vaccine to induce effective protection in cattle against *R. microplus* infestation. Although the precise function of Bm86 has not been described, the vaccine impairs the survival of ticks on immunized cattle and substantially reduces engorgement weights, which is caused by a disruption of the tick midgut epithelium, suggesting Bm86′s role in the development of tick midgut tissue during the engorgement process^14,15^. The Bm86-based vaccine can also affect the egg-laying capacities of surviving ticks. Notably, besides reducing tick populations, Bm86-based vaccines have the potential to partially block the transmission of tick-borne pathogens like *Babesia ovis*^16,17^. Despite their promise as anti-tick vaccines for cattle, vaccination with Bm86 orthologs in *I. ricinus* ticks (Ir86-1 and Ir86-2) did not show obvious effects on the feeding parameters of *I. ricinus*^18,19^, suggesting that certain species of ticks may be refractory to the Bm86 vaccine, and that immunization with the whole protein (which was the case with the *I. ricinus* orthologs) may induce immunodominant but non-neutralizing antibodies.

Many tick proteins, such as vitellogenin receptor^20^, *I. scapularis* Bm86 orthologs, and ATAQ proteins^13^, feature epidermal growth factor (EGF)-like domains, although their functions in vector biology remain elusive. The Bm86 antigens in particular feature multiple EGF-like domains^13^. Proteins harboring EGF-like domains have demonstrated the evolution of multiple distinct functions^21^. They are associated with stimulating cell growth and restoring membrane damage, in addition to supporting microbial virulence, such as the invasion of *Plasmodium falciparum* into erythrocytes^22^ and *Neisseria meningitidis*^23^ into endothelial cells. Recent studies have shown that a monoclonal antibody against an EGF-like domain of a *Plasmodium* protein prevented parasite invasion via inhibition of the pathogen’s erythrocyte-binding capacity^24^. In the fruit fly, the EGF receptor pathways control stem cell proliferation and gut remodeling following infection^25^. While multiple proteins with EGF-like domains from hard and soft ticks were identified^20,26^, their roles in vector physiology or development remain enigmatic. The tick gut presents a pivotal microbial entry point and serves as the major organ for pathogen colonization and survival within the vector, especially for the Lyme disease pathogen, as it resides exclusively in the tick gut^27,28^. Here we report that Bm86 orthologs in *I. scapularis* (Is86) are expressed in the tick gut and contain three EGF-like domains, and that immunization with recombinant EGF-like domains influences optimal blood meal engorgement and the molting of *I. scapularis*, in addition to partially blocking pathogen transmission from tick to host. These studies may help in the development of anti-tick vaccines to combat Lyme disease.

## Results

### Identification and expression of epidermal growth factor (EGF)-like domains in *I. scapularis*

**Figure 1:**
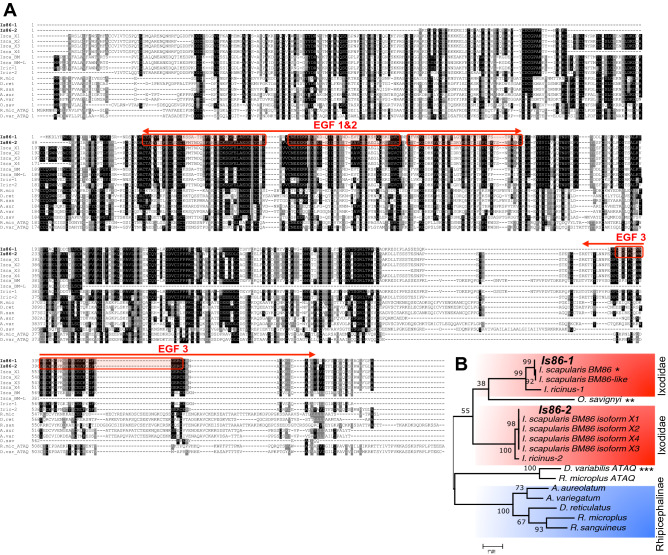
Multiple sequence alignment and phylogeny of Bm86 orthologs. (**A**) Alignment of orthologous Bm86 protein sequences in hard and soft ticks generated in Clustal Omega and BoxShade (https://www.ch.embnet.org/software/BOX_form.html). The letters with a black background are identical amino acids, while gray background letters are similar under the 50% majority rule. Red arrows span the amino acid sequences that were used to generate the recombinant EGF-like domains 1, 2, and 3, as denoted by the red boxes. EGF-1 and EGF-2 are *Is86-1* and *Is86-2* sequence-specific, respectively, while EGF-3 is sequentially identical for both *Is86-1* and *Is86-2*. (**B**) Phylogenetic relationship of Bm86 orthologs among hard and soft ticks based on maximum likelihood tree. The numbers by the nodes represent a percent support using 1000 bootstrap replicates. A single asterisk indicates the putative sequences of *Is86* recently added to the NCBI GenBank database, two asterisks indicate putative *Bm86* orthologs in the soft tick species *O. savignyi*, and three asterisks denote ATAQ and *Bm86*-related homologs.

As the Bm86 glycoprotein in cattle ticks has been used as a commercially available vaccine against tick infestation in cattle^17,29,30^, we sought to know if vaccination with the Bm86 ortholog in *I. scapularis* exerts similar effects. Using the NCBI BLAST program to compare sequences with *R*. *microplus* Bm86 and *I. ricinus* orthologs (Ir86), we noticed that there were multiple Bm86 transcript variants in *I. scapularis*, as shown in sequence alignments (Fig. 1A). Using specific primers (Table S1) and the *I. scapularis* cDNA template, two homologs termed as *Is86-1* and *Is86-2* were PCR amplified. Furthermore, Bm86 features at least three identifiable epidermal growth factor (EGF)-like domains, designated herein as EGF-1, EGF-2, and EGF-3 (Fig. 1A). The sequence analysis indicates that EGF-3 is identical for both *Is86* homologs, whereas EGF-1 and EGF-2 have sequence specificities for *Is86-1* and *Is86-2*, respectively, with a sequence similarity of 47.9% between the two domains (Fig. S1A). Next, we generated the recombinant versions of the EGF-1, -2, and -3 proteins in a bacterial expression system (Fig. S1B, upper panel). Antisera were also raised in mice that specifically recognized the corresponding recombinant EGF proteins (Fig. S1B, lower panel). The phylogenetic tree analysis (Fig. 1B), comparing Is86 with Bm86 orthologs from a representative set of other hard and soft tick species, demonstrated a clear orthology with Ir86 of *I. ricinus*, which is the closest relative among the tested tick species, and showed protein homologies of 88% and 44% for Is86-1 and Is86-2, respectively. The Bm86 of *R. microplus* clustered with other *Rhipicephalinae* tick species, such as *Dermacentor* and *Amblyomma*. Interestingly, a putative Bm86 ortholog from the soft tick species *O. savignyi* was subgrouped with Is86-1.

### *Borrelia burgdorferi* infection induces *Is86* expression in ticks

**Figure 2:**
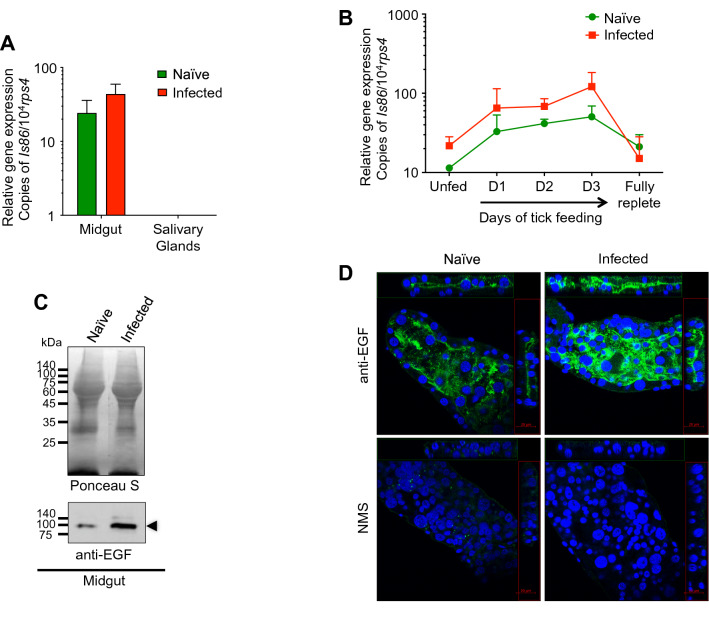
Expression and localization of *Is86* in *I. scapularis* ticks. (**A**) Gene expression of *Is86* in ticks. The naïve and *B. burgdorferi*-infected nymphal ticks were collected during feeding (at days 1, 2, and 3 of feeding, and when fully replete). The tick guts and salivary glands of unfed and fed ticks were dissected and pooled together according to tissue type. *Is86* gene expression was measured by using qPCR and normalizing against the tick house-keeping gene *rps4*. (**B**) Kinetic *Is86* expression over the course of tick feeding. Naïve and *B. burgdorferi*-infected ticks were collected at the indicated time points during feeding. *Is86* gene expression was measured by using qPCR and normalizing against tick *rps4*. The results are presented as the median ± SEM from two independent experiments. (**C**) Is86 protein expression is induced in *B. burgdorferi*-infected tick guts. The proteins from naïve and *B. burgdorferi*-infected nymphs (pooled unfed and two-day fed guts) were either stained with Ponceau S to show an equal protein loading (upper panel) or probed with pooled antisera against Is86 EGF domains (lower panel). The arrowhead indicates Is86 immunoreactivity, which is notably induced in spirochete-infected guts, in comparison to naïve tick guts. For an image of the full-length immunoblot, please refer to Fig.S6. (**D**) Is86 is localized towards the luminal surface of the unfed tick gut. Unfed tick guts were stained with anti-EGF (green) or normal mice serum (NMS), and the nuclei were stained with DAPI (blue) and visualized under the confocal microscope. The representative images are composites of Z-stack.

To explore the biological function of Is86 in *I. scapularis* and its roles in *B. burgdorferi* infection, we first investigated the expression of *Is86* in ticks with or without spirochete infection. Consistent with previous reports that the *Bm86* gene was expressed specifically in the tick gut with upregulation during feeding^13^, *Is86* was expressed predominantly in the *Ixodes* tick gut and was completely absent in the salivary glands. Furthermore, although not statistically significant, the mean *Is86* level in infected tick guts was 1.8-fold higher than in naïve ticks (Fig. 2A). We also assessed whether *B. burgdorferi* infection influences *Is86* expression kinetics during the course of tick feeding. The results showed that *Is86* expression was upregulated after the onset of tick feeding, with the level of expression steadily elevating and peaking at day three. Decreased expression was observed in post-fed ticks in a level comparable to that of unfed ticks (Fig. 2B), potentially because there is a reduced requirement for the protein following repletion, as it is possibly involved in the development and remodeling of the tick gut during feeding events. The level of *Is86* in *B. burgdorferi-*infected tick guts was constantly higher compared to naïve ticks during feeding (Fig. 2B). Consistently, a specific reaction around 100 kDa was detected in tick gut proteins using the pooled Is86 EGF antisera, and the protein level in infected tick guts was higher than in naïve ticks (Fig. 2C, Fig.S6). In order to support the specificity of the reaction, we isolated native Is86 from unfed adult tick guts using immunoprecipitation (Fig. S3, left panel), which specifically reacted with EGF antisera at a molecular weight of about 100 kDa (Fig. S3, right panel). Similarly, immunofluorescent staining showed that a specific reaction was detected in both naïve and *B. burgdorferi*-infected tick guts (Fig. 2D). Although our study used permeabilized gut samples, the predominant distribution of immunofluorescent signals towards the luminal side of the *Ixodes* gut is consistent with the prevailing notion that *Bm86* is expressed on the surface of the tick gut epithelial cells^13,31^. Furthermore, an apparent higher fluorescence was observed in infected tick guts, as compared to naïve ticks (Fig. 2D). Taken together, these results indicate that *B. burgdorferi* infection could upregulate *Is86* expression.

**Figure 3:**
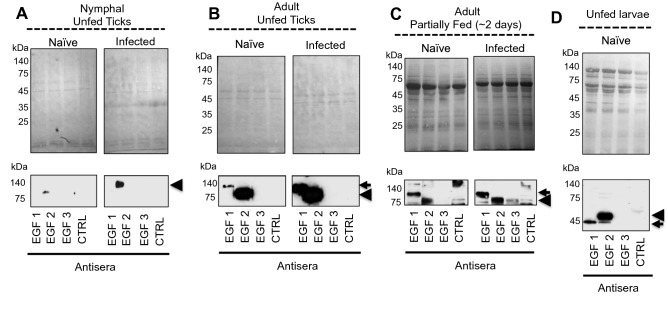
Expression of Is86 homologs in various tick stages. Nymphs were allowed to feed on naïve or infected mice and collected after 48 h of feeding. The naïve and infected adult ticks fed on an artificial feeding system and were collected after 48 h of feeding. The unfed nymphal ticks (**A**), as well as the dissected guts from (**B**) unfed adult ticks, (**C**) partially-fed (two-day) adult ticks, and (**D**) unfed larval ticks, were processed using SDS-PAGE, transferred to nitrocellulose membrane, stained with Ponceau S to show an equal protein loading (upper panel), and probed with antiserum against each Is86 EGF domain (EGF-1, -2, or -3). PBS and adjuvant-immunized sera were used as controls (CTRL). Arrows indicate the specific antibody response to Is86-1, and arrowheads indicate the specific response to Is86-2. For an image of the full-length immunoblot, please refer to Fig. S7.

Next, we tested which Is86 homologs (Is86-1 or Is86-2) were expressed temporally in ticks at various stages of the life cycle. Since EGF-1 and EGF-2 are predominantly represented in Is86-1 and Is86-2, respectively, we used antiserum against each EGF domain. EGF-2 was detected in unfed nymphal ticks (Fig. 3A), while both Is86 homologs were detected in unfed (Fig. 3B) and partially fed (Fig. 3C) adult tick guts. Protein levels were upregulated after *B. burgdorferi* infection (Fig. 3A–C). The homologs displayed the same migrating patterns and sizes in both nymphal and adult ticks. In silico analysis revealed various patterns and types of posttranslational modifications (Fig. S2). Interestingly, analysis of the unfed naïve larvae revealed the presence of both Is86 homologs migrating with the predicted molecular masses of 44.2 kDa (Is86-1) and 50.5 kDa (Is86-2) (Fig. 3D), suggesting an absence of posttranslational modifications of the protein homologs in larvae prior to blood meal engorgement. The testing of Is86 homolog expression in fully replete naïve and infected larvae failed, likely due to the blood content in the tested samples or the lack of protein expression (data not shown). Additionally, we did not detect any specific reactions in any stage of ticks using EGF-3 antiserum, suggesting that either the level of EGF-3 is low, or that the antigen does not contain an immunogenic epitope that could be recognized by the specific antiserum (Fig. 3, Fig. S7).

### EGF-like domain antibodies marginally interfere with tick physiology and affect *B. burgdorferi* transmission by ticks

**Figure 4:**
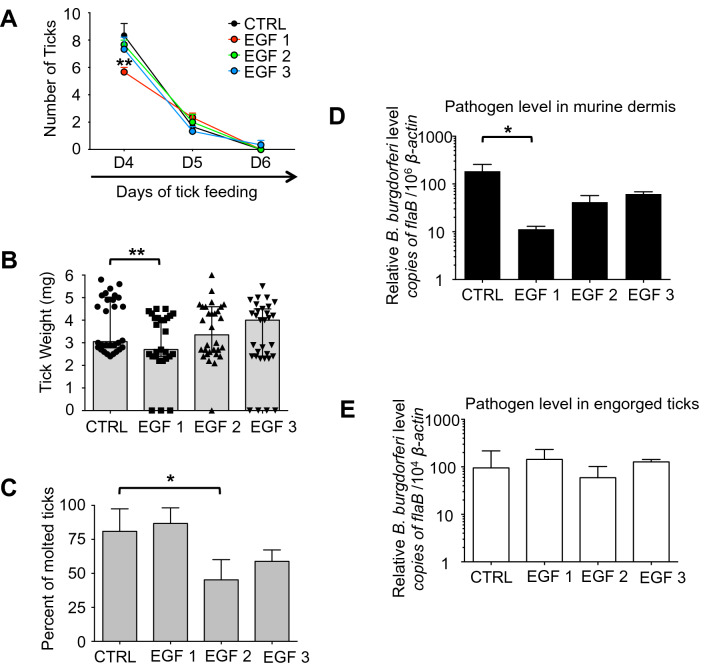
Immunization with EGF domains affects tick feeding and interferes with tick transmission of *B. burgdorferi*. *B. burgdorferi*-infected ticks were allowed to feed on mice immunized with Is86 EGF-1, -2, or -3. Ticks that fed on mice immunized with adjuvant only were used as controls. The parameters of tick feeding were recorded. (**A**) Mean number of replete ticks, with error bars showing standard error of mean (± SEM). Fully replete ticks were collected and counted (n = 30, 24, 29, 27/group, respectively). The amount of EGF-1 ticks collected on day 4 was significantly lower (**p < 0.01). (**B**) Median tick engorgement weight, with error bars showing 95% confidence intervals (CI). The weights of individual fully replete ticks (n = 30, 27, 30, 30/group, respectively) were measured. Data points with zero values indicate unattached live or dead ticks collected 24 h post infestation. Fully replete EGF-1 ticks weighed significantly less (**p < 0.01). (**C**) Mean tick molting rate, with error bars showing ± SEM. The collected ticks were allowed to molt (n = 21, 15, 20, 17/group, respectively) in the incubator, and the percentage of molted ticks was calculated. The asterisks indicate statistically significant differences at p < 0.05. (**D**) Mean *B. burgdorferi* burden in mouse skin. This experiment represents the transmission of spirochetes from infected ticks to naïve mice. Murine skin samples were collected 10 days after tick feeding. Spirochete levels were evaluated by measuring copies of *flaB* using qPCR and normalizing against mouse *β-actin*. The *B. burgdorferi* burden was significantly reduced in mice immunized with EGF-1, as compared to controls (*p < 0.05). Bars represent the mean ± SEM of four qPCR analyses of *B. burgdorferi* levels from two independent animal experiments. (**E**) Median spirochete burden in ticks (n = 3/group), with error bars showing 95% CI. Spirochete burdens in fully replete ticks were assessed by measuring copies of *flaB* using qPCR and normalizing against tick *β-actin.* There was no difference among all EGF domain-immunized and control groups.

Previous studies have shown that specific antibodies against Bm86 affect tick feeding on cattle^17,32^. Despite promising activity as anti-tick vaccines, vaccination with the Bm86 orthologs in *I. ricinus* (Ir86-1 and Ir86-2) did not show obvious effects on tick feeding^18^. We hypothesize that instead of immunization with the whole protein, which may induce immunodominant but non-neutralizing antibodies, identifying and focusing on the most conserved region of Is86 may produce the most useful and effective neutralizing antibodies. We sought to know whether EGF-like domain-specific antibodies targeting the Is86 homologs could impair tick physiology and consequently *B. burgdorferi* persistence and transmission by ticks. Groups of mice were immunized with individual Is86 EGF-1, -2, or -3, which elicited high titers of antibodies against the Is86 homologs, in contrast to the controls (Fig. S4A). The mice were then infested with infected nymphs (10 ticks/mouse), and feeding parameters were observed. Compared to the controls, significantly less fully replete ticks were collected from mice immunized with Is86 EGF-1 (Fig. 4A, p < 0.01), and the engorgement weights of the ticks were substantially lower (Fig. 4B, p < 0.01). Such data were not observed after immunization with Is86 EGF-2 or -3, suggesting that antibodies against Is86 EGF-1 could interfere with tick physiology and result in a delay of tick feeding. Additionally, several unattached live and/or dead ticks were observed after feeding on mice in the experimental groups, which did not occur in the controls (Fig. 4B). The collected ticks were then stored in the incubator and allowed to molt. The percentage of molted ticks that had fed on mice immunized with EGF-2 was significantly decreased, with a molting rate of 40 ± 8.5%, as compared to the controls (81 ± 9.5%) (Fig. 4C, p < 0.05). As Bm86-based vaccines have been reported to partially block the transmission of tick-borne pathogens like *B. ovis*^17^, we also assessed the effects of EGF antibodies on *Borrelia* transmission by ticks. Ten days after tick feeding, murine skin samples were collected. The spirochete burden in mice immunized with EGF-1 was significantly decreased compared to the controls (Fig. 4D, p < 0.05). This was not caused by disparities in spirochete burdens within the ticks (Fig. 4E), indicating that antibodies against the Is86 homologs do not impact the majority of spirochetes that persist in ticks, but potentially influence the dissemination of a fraction of *B. burgdorferi* through ticks.

**Figure 5:**
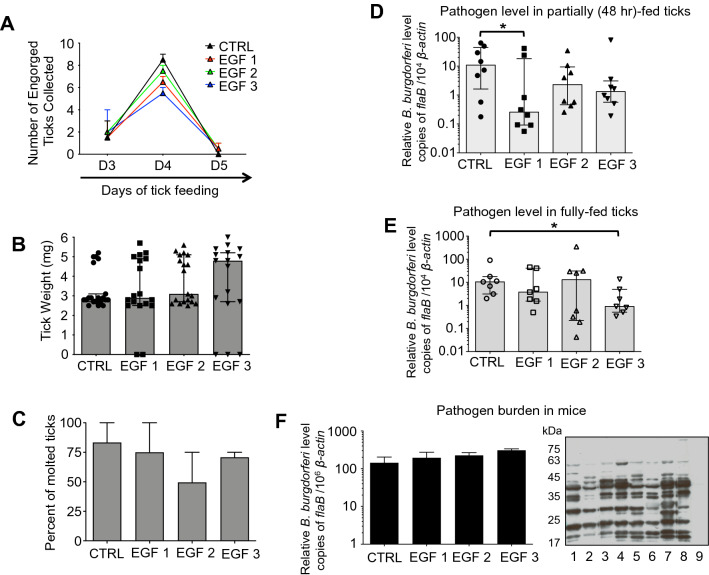
EGF immunization affects *B. burgdorferi* acquisition. Mice were immunized with Is86 EGF-1, -2, or -3, infected with *B. burgdorferi*, and then parasitized by naïve ticks. Ticks that fed on mice immunized with adjuvant only were used as controls. (**A**) Mean number of replete ticks, with error bars showing ± SEM. Fully replete ticks were collected and counted (n = 20, 17, 20, 16 ticks/group of 2 mice, respectively). (**B**) Median tick engorgement weight. The weights of fully replete ticks (n = 20, 18, 19, 17 ticks/group, respectively) were measured with error bars showing 95% confidence intervals (CI). Data points with zero values indicate unattached live or dead ticks collected 24 h post infestation. (**C**) Mean tick molting rate, with error bars showing ± SEM. The collected ticks were allowed to molt (n = 6, 7, 8, 7 ticks/group of 2 mice, respectively) in the incubator, and the percentage of molted ticks was calculated. Median *B. burgdorferi* burdens in (**D**) 48-h fed ticks and (**E**) fully replete ticks (n = 8, 7 ticks/group, respectively), with error bars showing 95% CI. Spirochete burdens in ticks were assessed by measuring copies of *flaB* using qPCR and normalizing against tick *β-actin.* (**F**) Mean *B. burgdorferi* burden in mouse skin (n = 2), with error bars showing ± SEM. This experiment represents the acquisition of spirochetes from infected mice to naïve ticks. Murine skin samples were collected after the completion of tick feeding. Spirochete levels were evaluated by measuring copies of *flaB* using qPCR and normalizing against mouse *β-actin*. The right-side panel shows a Western blot of *B. burgdorferi* lysates, which were probed with *B. burgdorferi*-infected mouse sera (control groups in lanes 1 and 2, EGF-1-immunized in lanes 3 and 4, EGF-2-immunized in lanes 5 and 6, EGF-3-immunized in lanes 7 and 8, and normal mouse serum in lane 9). The asterisks in the graphs indicate statistically significant differences at p < 0.05.

We then assessed whether antibodies against the EGF domains interfere with spirochete acquisition by ticks. Mice were immunized with individual EGF-1, -2, or -3 (Fig. S4B), and then infected with *B. burgdorferi* via needle inoculation. Twelve days after inoculation, naïve ticks were allowed to feed on the mice. There were no significant differences in tick feeding duration (Fig. 5A) and tick engorgement weights (Fig. 5B) among the experimental and control groups. Although not statistically significant, there was an apparent effect on molting success, as the mean percentage of adult ticks that molted from engorged nymphs was reduced after feeding on EGF-2 immunized mice (50 ± 26%), as compared to control ticks (83 ± 17%) (Fig. 5C). Notably, after 48 h of feeding, the spirochete level in ticks that parasitized EGF-1 immunized mice was 44-fold lower (Fig. 5D, p < 0.01), and 4.7-fold and 8.3-fold lower in the EGF-2 and EGF-3 groups, respectively (Fig. 5D), in comparison to the controls. However, in fully replete ticks, only those that fed on EGF-3 immunized mice displayed a significant decrease in spirochete burden, when compared to control ticks (median of 11.6-fold, p < 0.05) (Fig. 5E). The impairment of spirochete survival in fed ticks was likely due to the neutralizing effects of anti-EGF antibodies in the ticks, but not in the murine hosts, as similar levels of *B. burgdorferi* were detected in the mouse dermis, from which the pathogen likely enters the vector (Fig. 5F). Despite a partial impact on spirochete persistence (Fig. 4E), *B. burgdorferi* transmission from tick to host was not affected, and comparable levels of spirochetes were detected in the mouse skin among all groups (Fig. 5F). As naïve and *B. burgdorferi*-infected ticks represent two different environments physiologically, where the expression of *Is86* (Fig. 2) and EGF immunoreactivity (Fig. 3, Fig. S6) vary, we performed an independent rEGF immunization experiment to investigate the potential impact of anti-rEGF antibodies on the physiology of ticks without *B. burgdorferi* infection. In contrast to infected ticks, rEGF immunization did not significantly impact the feeding or molting parameters of naïve ticks (Fig. S5), likely due to the dramatically reduced production of Is86 antigens in naïve ticks (Fig. 3, Fig. S6).

### *Is86* silencing failed to interfere with *B. burgdorferi* persistence and transmission

**Figure 6:**
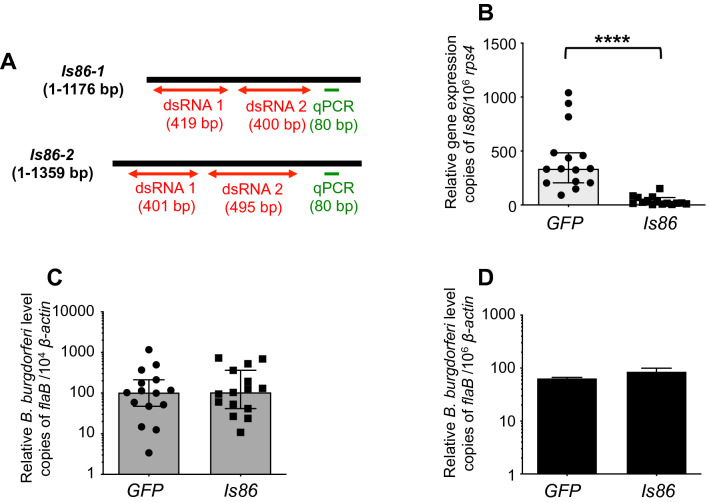
Silencing of *Is86* fails to interfere with *B. burgdorferi* persistence in ticks and spirochete transmission. (**A**) Schematic representation of the full open reading frame of *Is86-1* and *Is86-2*, showing regions targeted for RNA interference. The regions encompassing dsRNA constructs (red arrows) and detection primers that contain common sequences in both *Is86-1* and *Is86-2* (green) are shown. (**B**) RNAi induced a significant knockdown of *Is86* transcripts (****p < 0.0001). The levels of *Is86* gene transcripts in ticks that had been microinjected with pooled dsRNAs targeting *Is86-1* and *Is86-2* were significantly decreased compared to the ds*GFP* RNA control. (**C**) Silencing of *Is86* did not affect spirochete persistence in ticks. Spirochete burdens in fully replete ticks were assessed using qPCR and normalizing against tick *β-actin*. (**D**) Silencing of *Is86* did not block *B. burgdorferi* transmission from ticks. Spirochete burdens in murine skin were assessed using qPCR. The bars represent the mean ± SEM of four qPCR analyses of *B. burgdorferi* levels, derived from two independent animal experiments.

To directly assess the role of Is86 proteins in tick physiology and *B. burgdorferi* infectivity, we employed an RNA interference-mediated knockdown of *Is86* in ticks. To efficiently knock down gene expression, two dsRNA constructs targeting different regions of *Is86-1* and *Is86-2* were generated (Fig. 6A). *B. burgdorferi*-infected nymphs were microinjected with the pooled ds*Is86* RNAs, targeting both *Is86* homologs, and allowed to feed on naïve mice. Compared to ticks injected with ds*GFP* RNA, *Is86* transcripts in fully replete ticks were significantly decreased after treatment with ds*Is86* RNAs (Fig. 6B). However, the silencing of *Is86* did not affect *B. burgdorferi* persistence in ticks (Fig. 6C). Ten days after tick feeding, mouse skin samples were collected. Comparable spirochete levels were detected in mice that had been parasitized by ticks injected with ds*Is86* RNAs and ds*GFP* RNA (Fig. 6D). The results indicated that the RNAi-mediated knockdown of *Is86* failed to influence spirochete persistence in ticks and pathogen transmission to the host.

## Discussion

Many tick proteins, including ones from *I. scapularis*, feature one or multiple EGF-like domains, although their functions in vector biology or pathogen persistence remain elusive. The Bm86 glycoprotein, originally isolated from *R. microplus* ticks^10,33,34^, represents the only tick antigen to be commercially developed as an anti-tick vaccine, protecting immunized cattle from tick infestation. The vaccine is also partially effective in blocking the transmission of certain tick-borne pathogens, such as *Babesia* parasites^17,35^. Although this study lacks conceptual innovation, as Bm86 orthologs have been subjected to many earlier studies, including the vaccination of *Ixodes* species^13^ with Bm86 homologs, which failed to influence *I. ricinus* feeding or oviposition^18^, our current study identified a biological significance of the EGF domains in *Ixodes* ticks. Particularly, we discovered that immunization with specific EGF-like domains in the Bm86 orthologs of *I. scapularis* (originally identified by Nijhof and colleagues^13^,) could impact, at least to a marginal extent, tick feeding and molting success, as well as the survival of *B. burgdorferi* in the vector, highlighting their roles in tick biology and suggesting their use as potential components of anti-tick vaccines.

Based on the available *I. scapularis* genome data^36,37^, we identified at least two homologs of *Bm86* (*Is86-1* and *Is86-2*); however, according to further updates in the NCBI database, these homologs likely incorporate additional members, including several splice variants. Nonetheless, our sequence alignment and phylogenetic analysis revealed that *Is86-1* and *Is86-2* represent prototypes of two major groups of *Bm86* orthologs in black-legged ticks, which is in agreement with a previous report^13^ that showed a divergence between these *Is86* homologs from prostriata ticks to other hard tick species in the metastriata group. Notably, the relatively low sequence identity of *Is86* homologs when aligned to the closely related *I. ricinus Ir86* further underscores its extensive sequence diversification, even across closely related tick species. Such sequence variability, which is also observed for *Bm86* in *Rhipicephalus (Boophilus)* spp. ticks^38^, may reflect their functional diversity among various tick species. Although the exact function of the Bm86 protein family still remains highly enigmatic, these gene-products feature several conserved domains that are typically found in the epidermal growth factor (EGF) family of proteins^13,15^. The EGF domains are usually represented as a small domain of 30–40 amino acids which regulate a diverse array of cellular functions, primarily supporting the growth and development of an organism^14,21^. In our present study, we identified at least three EGF-like domains in Is86 homologs in *I. scapularis*, although additional EGF domains have been reported to occur in various *Ixodes* spp. ticks^13^.

An important role of Bm86 in the physiology of *R. microplus* ticks, as well as in the transmission of *Babesia* species in cattle, was previously reported^17^. In fact, vaccines based on Bm86 antigens have proven to be a feasible control method against *R. microplus* tick infestations in multiple countries. However, primarily due to the extensive sequence diversity across tick species, Bm86 orthologs are likely to undergo dramatic functional divergence, thereby lacking broad vaccine efficacy in many other tick species^32^. In fact, an amino acid sequence divergence of greater than 2.8% could result in a decreased vaccine efficiency of Bm86 antigens^39^. Accordingly, as with *I. ricinus* ticks, vaccination with Bm86 homologs failed to influence tick feeding or oviposition^18^. These findings agree with the results of our RNAi-mediated gene silencing studies, as the knockdown of Is86 homologs did not impact *I. scapularis* physiology or pathogen survival. The silencing of the *R. microplus Bm86* gene via RNAi also failed to affect the efficiency of the transovarial transmission of *B. bovis*^40^. Nevertheless, despite an absence of gene silencing effects, the partial impact of immunization with the truncated Is86 antigens suggests the potential roles of the EGF-like domains in tick biology. The importance of these domains in Is86 (or similar antigens) in regards to tick biology is particularly highlighted by: (1) their induction during the tick engorgement process, and more importantly during the *B. burgdorferi* infection of ticks, (2) their extracellular exposure, in particular towards the luminal surfaces of the midgut cells, and consequent antibody accessibility, (3) their copious expression throughout the tick developmental stages, including sub-adult and adult, (4) the abilities of anti-EGF antisera to impact, at least marginally, multiple aspects of tick biology, including blood meal engorgement and molting, and finally (5) their detectable influences on spirochete survival in ticks, in terms of both pathogen acquisition and transmission through the vector to the host. In order to expand upon these promising initial results, the effect of EGF immunization on *Borrelia* transmission requires more elaborate studies to better address the vaccine potential of these antigens. As Bm86 orthologs bear considerable sequence homology to closely related ticks, such as *I. ricinus*, similar studies would shed light on the potential applicability of EGF domains as global vaccines.

Despite our identification of the EGF-like domains in Is86 antigens as a possible component of anti-tick vaccine targets, we were unable to decipher their precise biological functions. Vaccination against its ortholog, Bm86, reportedly resulted in tissue damage in the midgut of adult *B. microplus* ticks^41^. Based on our data, no such effect was apparent in *I. scapularis* nymphal ticks, either via anti-Is86 immunization or through RNAi-mediated gene silencing. It is therefore possible that the anti-Is86 antibodies are cross-reactive to other unknown tick proteins, and that the observed vaccination effects of EGF-like domain antibodies are impacted by their recognition of other cross-reactive antigens, rather than the specific inhibition of Is86 homologs. Although our immunoblot and immunoprecipitation studies using anti-EGF antibodies failed to identify additional tick proteins other than Is86, these antibodies still can bind to and inhibit the activity of unidentified tick gut proteins through steric hindrance^42^, which could play essential supportive roles in tick biology and pathogen survival.

We noticed perplexing discrepancies in the abilities of specific EGF antisera to detect target proteins at various tick feeding or developmental stages. Although Bm86 orthologs are known to be glycosylated, our study recorded a puzzling and substantially higher molecular weight of both Is86 orthologs. Some tick physiological parameters were affected to some extent for both homologs, but more significant results were observed with anti-EGF-1 antibodies (Is86-1). While the expression patterns of the homologs were comparable towards the end of tick feeding, differences were noted in unfed and early-fed ticks, with Is86-1 being absent in unfed tick guts and generally being expressed at lower levels than Is86-2. It is also possible that, in addition to or in lieu of Is86, the EGF antibodies also detected multiple patterns of Is86 glycosylation, splice variants, or cleavage products, or recognized additional cross-reactive tick antigens, that are differentially produced during tick feeding or the development process. Nevertheless, we observed that Is86-1 contained a broader spectrum of posttranslational modifications at a higher frequency than Is86-2. Such differences may be a contributing factor as to why the antibody-based blocking of Is86-1 caused physiological changes in ticks, such as delayed feeding and reduced weight, indicating a dominant physiological function of Is86-1. Although marginal, as EGF-1 and EGF-2 antibodies affected distinct phases of tick biology, namely blood meal engorgement and the molting process, respectively, it is likely that both Is86 homologs, or other tick gut protein(s) with cross-reactive EGF antibodies, have predominant functions at distinct phases of the tick life cycle. Our study also uncovered a novel mode of action for the EGF-based antigens, suggesting that other unknown molecules are implicated in the process. This may also include tick molecules that are induced during borrelial infection, and/or the spirochete proteins that interact with the gut surface receptors and the antibodies targeting EGF-like domains, which interrupt that interaction.

There has been an increasing body of evidence that EGF-like domains are involved in versatile functions during cellular growth and development^21^, including protein–protein interactions^43^ and cellular adhesion with concomitant intracellular signaling^44^. In particular, due to the well-documented roles of EGF proteins in cell growth, proliferation, repair, or remodeling, as well as infection control^22,23,25^, it is likely that the EGF domains in Is86 proteins may serve critical roles in *Ixodes* biology and infection. We speculate that the induction of Is86 during blood meal engorgement, or more notably during infection, could involve discrete protein–protein interactions relevant to *B. burgdorferi*, either directly via EGF domain interactions with spirochetes, or indirectly via another EGF domain-containing protein. This possibility is bolstered by the fact that EGF-like domains in Is86, or in other cross-reactive proteins, are exposed predominantly at the luminal side of the tick gut epithelium (a localization pattern also reported for Bm86^14^), where extracellular pathogens like *B. burgdorferi* reside and express a plethora of surface antigens, many of which are known to be involved in host–pathogen interactions^27,45^. A deeper understanding of the biological functions of the Is86 homologs and their roles in *I. scapularis* tick physiology, as well as the precise molecular mechanisms through which antibodies against EGF-like domains or other novel tick antigens of unknown functions reduce spirochete entry and exit through ticks, will not only enrich our knowledge of tick-pathogen interactions, but will also ultimately impact the development of new strategies for the prevention of tick-transmitted infections.

## Methods

### Bacteria, mice, and ticks

*Borrelia burgdorferi* sensu stricto infectious isolate B31 A3, grown in Barbour-Stoenner-Kelly-H (BSK-H) medium, was used throughout this study^46^. Four- to six-week-old C3H/HeN mice were purchased from the Charles River Laboratories. All experimental protocols were approved and performed in accordance with the guidelines of the Institutional Animal Care and Use Committee and Institutional Biosafety Committee of the University of Maryland, College Park. The study was carried out in compliance with the ARRIVE guidelines. *Ixodes scapularis* tick egg masses were purchased from the Oklahoma State University Tick Rearing Facility. Larval ticks were allowed to feed on naïve or *B. burgdorferi*-infected mice^47^; upon engorgement, the ticks were collected and maintained in an incubator at 20 °C with 95% relative humidity and a 12-h light/dark photoperiod regimen. The collected larvae were allowed to molt to nymphs, which typically took 4 weeks. The unfed nymphs were then allowed to parasitize mice; when fully replete, the ticks were collected and allowed to molt to adult ticks in the incubator.

### Polymerase chain reaction (PCR)

The oligonucleotide sequences for each of the primers used in specific PCR reactions are listed in Supplementary Table S1. The pathogen-free naïve and *B. burgdorferi*-infected unfed and fed nymphal ticks were collected at various time points during feeding (Days 1, 2, or 3, or as fully replete ticks). The tick specimens were immobilized, and the midgut and salivary glands were dissected. To increase the sensitivity of *Is86* detection in our initial experiments, we pooled tick samples from various time points of feeding and used a generic qPCR primer pair that could detect other *Is86* transcripts. Additionally, groups of naïve or *B. burgdorferi*-infected mice were parasitized by naïve or infected ticks, which were allowed to feed to full repletion, and murine skin biopsies were collected ten days after the ticks dropped off. Due to challenges associated with their size, larval and nymphal samples were processed differently. Nymphs were dissected for the isolation of specific organs (such as the gut or salivary glands), whereas larval samples were processed as entire bodies. Total RNAs were isolated either from tick or mouse tissues using TRIzol (Invitrogen), reverse transcribed to complementary DNA (cDNA) (Invitrogen), and treated with DNase (NEB) to minimize DNA contamination as detailed^48^.The gene transcripts were analyzed using quantitative PCR (qPCR). The amplification parameters for *rps4*/*Is86* are as follows: initial denaturation at 95 °C for 5 min, followed by 40 cycles each at 95 °C for 10 s, 55 °C for 20 s, and 72 °C for 30 s. The amplification parameters for tick *β*-actin/*flaB* are as follows: initial denaturation at 95 °C for 5 min, followed by 40 cycles each at 95 °C for 10 s and 60 °C for 1 min. The final step in both amplification cycles was the melt curve analysis at 55 °C for 30 s, increased by 0.5 °C per cycle to 95 °C. The amplification was performed in an iQ5 real-time thermal cycler (Bio-Rad) using SYBR Green Master Mix (Thermo Fisher Scientific). The relative transcript levels of *Is86* were measured against the tick house-keeping gene *rps4* in a three-step amplification cycle using annealing temperatures of 55 °C as detailed^49^. The relative spirochete burdens were assessed by measuring copies of *flaB* transcripts as a better surrogate for live pathogens^48^ in a two-step amplification cycle, using annealing temperatures of 60 °C and normalizing against the *β-actin* gene as detailed^48^.

### Generation of recombinant proteins and polyclonal antisera

To identify the *Bm86* ortholog in the *I. scapularis* genome, the *Bm86* genes from *R. microplus* and *I. ricinus* were used for similarity and identity comparison against the *I. scapularis* genome, which is available from the NCBI database (XM_029991986.1). Two *Bm86* homologs, *Is86-1* and *Is86-2*, were PCR amplified using a template from the *I. scapularis* genome^37^, cloned into the pGEM-T Easy Vector (Promega), sequenced, and deposited in the NCBI nucleotide database. The EGF-like domains within *Is86-1* and *Is86-2* were predicted according to published data^15^, and further validated using the PROSITE server (https://prosite.expasy.org). Clones harboring either *Is86-1* or *Is86-2* were used as templates for further PCR amplification of EGF-1 and EGF-2, respectively, using primers containing restriction sites (Table S1). EGF-3 was amplified from both clones. The PCR-amplified EGF domain products (EGF-1, -2, and -3) were subcloned into a pET28a expression vector. Heterologous expression of the three recombinant EGF-like domains was induced in BL21 (DE3) *E. coli* with 0.4 mM IPTG at 37 °C. The recombinant proteins, containing 6xHis-tag located at the N-terminus, were purified using ProBond Nickel-Chelating Resin (Thermo Fisher Scientific), dialyzed, and refolded in 50 mM Tris–HCl. Polyclonal antisera against each of the three recombinant domains were generated in mice as described^50^. The antibody titers were assessed using an enzyme-linked immunosorbent assay (ELISA) and the specificity was evaluated using Western blotting^50^.

### Phylogenetic analysis

The protein sequences of *Bm86* orthologs among hard and soft tick species were based on multiple sequence alignments generated with ClustalW. The phylogenetic tree was constructed using the maximum likelihood method with 1000 bootstrap replicates in MEGA7 software^51^.

### Western blotting

Immunoblotting was performed as described^48^. Briefly, the dissected guts of unfed ticks (~ 30 μg of total larval lysate, 5 nymphs/lane, 3 females/lane) and two-day fed ticks (~ 30 μg of female gut lysate/lane, 2 nymphs/lane) were pooled, extracted and resolved by SDS-PAGE, and immunoblotted using antiserum (1:1,000) against Is86 homologs. The blots were developed by the addition of horseradish peroxidase (HRP)-conjugated secondary antibodies (1:5,000 to 10,000), using the chemiluminescent immunoblotting detection reagent (Thermo Scientific Inc.). Immunoblotting to confirm *B. burgdorferi* infection in mice was performed as described^48^.

### Active immunization with recombinant EGF domains and infection studies

For *B. burgdorferi* transmission experiments, mice were immunized with individual recombinant Is86 EGF-1, -2, or -3, as detailed^42,47^. Briefly, 10 μg of recombinant protein was dissolved in 50 μl of phosphate-buffered saline (PBS), emulsified with 50 μl of complete (first injection) or incomplete (remaining two injections) Freund's adjuvant, and subcutaneously administered into each mouse at 10-day intervals. As recombinant EGF proteins are relatively pure (Fig. S1B), mice immunized with PBS and adjuvant served as the only control group. Two weeks after the second boost, groups of mice (3 mice/group) were infested with *B. burgdorferi*-infected *I. scapularis* nymphs (10 ticks/mouse). A portion of ticks was forcibly detached after 48 h of feeding, and others were collected when they dropped off after full repletion. The spirochete burdens in 48-h fed and replete ticks were assessed using qPCR. Ten days after tick feeding, mice were euthanized, and skin biopsy samples were collected to assess the spirochete burdens by qPCR.

For *B. burgdorferi* acquisition experiments, mice were immunized with recombinant EGF-1, -2, or -3, as described in the above paragraph. The mice were then infected with *B. burgdorferi* via intradermal needle inoculation. Two weeks after inoculation, the mouse sera were collected and probed with *B. burgdorferi* lysates and immunoblotted to confirm infection, prior to infestation with naïve nymphs (15 ticks/mouse, 2 mice/group). Ticks were collected after 48 h of feeding and after full repletion. Spirochete burdens in the ticks were evaluated with qPCR, as described previously^42^. Following the completion of tick feeding, murine skin samples were collected to assess spirochete burdens using qPCR. In both infection studies, to monitor the progress of feeding, ticks were checked daily until all had detached from the mice. Detached ticks were collected and counted, and their engorgement weights were measured using a laboratory scale. Ticks were then allowed to molt, and their molting rates were calculated two months after repletion.

### RNA interference and infection studies

RNA interference (RNAi) experiments targeting tick *Is86-1* and *Is86-2* were conducted as described^42^. Briefly, the targeted sequences in *Is86-1* and *Is86-2* were PCR amplified using T7 promoter sequence-containing primers (Table S1). A fragment of the *GFP* gene was amplified as a control. The dsRNAs were synthesized and purified using the MEGAscript RNAi Kit (Ambion). To assess whether the silencing of *Is86* expression affects *B. burgdorferi* transmission by ticks, infected nymphs were microinjected with ds*Is86* RNA (pooled dsRNAs targeting both homologs) or ds*GFP* RNA (5 µg/µl) using a microinjector (Eppendorf). After overnight incubation, the ticks were allowed to feed on naïve mice until full repletion (10 ticks/mouse). The ticks were collected and individually processed for the assessment of gene silencing, using primers that bind further upstream and downstream of the target dsRNA sequence (Table S1). Mouse skin samples were collected 10 days after tick feeding. Pathogen levels in the individual ticks and in murine tissues were evaluated by measuring the *flaB* transcripts with qPCR and normalizing against tick and mouse *β-actin*, respectively.

### Artificial membrane tick feeding system

An artificial membrane feeder was used to generate adult ticks from naïve and *Borrelia*-infected nymphs. The system was developed using published procedures as detailed^52^. Although various *B. burgdorferi* sensu lato spirochetes can resist or remain sensitive to killing by the host complement, we had previously shown that commercial defibrinated bovine blood can used in our membrane feeder system to study the acquisition and transmission of *B. burgdorferi* B31 strain. Briefly, defibrinated bovine blood was used, and the blood was changed every 12 to 14 h. Adult ticks (10 ticks/capsule) were placed on the artificial membrane feeding system, allowed to feed on bovine blood until partial repletion (~ 48 h), and collected to process for further analysis. Adult ticks were used for Is86 expression and immunoprecipitation experiments.

### Immunoprecipitation

Native Is86 was immunoprecipitated from adult tick guts using the Protein G Immunoprecipitation Kit (Sigma-Aldrich) as detailed^53^. Briefly, a RIPA buffer (Sigma) served to extract a midgut lysate from 10 ticks (~ 10 µg), which was then incubated with the pooled EGF-1, -2, and -3 antisera. The immunoprecipitated products were resolved using SDS-PAGE and either stained with Sypro Ruby or probed with pooled EGF antisera.

### Confocal microscopy

Confocal microscopy was performed as detailed^42^. Unfed tick guts were fixed in 4% paraformaldehyde at 4 °C overnight, then rinsed three times with PBS and permeabilized with acetone for 10 min. The tick tissues were blocked with 5% normal goat serum in PBS for 1 h at room temperature (RT) and then incubated with pooled EGF antisera at 4 °C overnight. After three washes with PBST (PBS with 0.05% Tween20), the tissues were incubated with Alexa Fluor 488-labelled goat anti-mouse IgG (Molecular Probes) for 1 h at room temperature, and the nuclei were stained with DAPI dye (Invitrogen) and imaged by a LSM510 laser confocal microscope (Zeiss), using the same fluorescence threshold setup for both naïve and *Borrelia*-infected samples as described previously^42^.

### Statistical analysis

The data were presented as median values with error bars indicating 95% confidence intervals (CI), or as mean values with error bars indicating the standard deviation (SD) or the standard error of the mean (SEM). Statistical differences were measured by using the non-parametric Mann Whitney two-tailed test or two-way ANOVA with Bonferroni post-tests using Prism 7 (GraphPad Software, Inc.).

## Supplementary Information

Supplementary Information
