# Supplementary material for: Antibodies against EGF-like domains in Ixodes scapularis BM86 orthologs impact tick feeding and survival of Borrelia burgdorferi
Source: Sci Rep. 2021 Mar 17;11:6095. doi: 10.1038/s41598-021-85624-5 (PMC7971074; doi:10.1038/s41598-021-85624-5)
Supplement: Supplementary file 1 — Supplementary Information [file 41598_2021_85624_MOESM1_ESM.pdf]

Supplementary materials

Antibodies against EGF-like domains in *Ixodes scapularis* BM86 orthologs impact tick feeding and survival of *Borrelia burgdorferi*

Juraj Koči1, Sandhya Bista, Payal Chirania, Xiuli Yang, Chrysoula Kitsou, Vipin Singh Rana, Ozlem Buyuktanir Yas, Daniel E. Sonenshine and Utpal Pal

Contains Figures S1, S2, S3, S4, S5, S6, and S7; legends to supplementary figures and Table S1

Fig. S1

A

EGF-1

M**CTAKVADPLCPNGCKSSATDLPFQCICKKGFQLAEDGITC**TERRV
CNEEEKKRCRKDQICGIVNDEVTCSCKSHQQEKDGVCTNE
CFVKKCSDPFANCEVYLGTEKCFCTRPLFSTRNITC*

EGF-2

M**CTANVTSPICPEGCTPMTNDQMPFHCKCKSGFELAEDGLTC**REKVV
CNEEEKKKCHESEICTIIDTKAVCTCAEGLQRLNGVCTDK
CTTKCDHKFAKCVIIDRYERCTCIYPLSKTSNGNQMC*

EGF-3

MTTTLNNPEDP**CISIPCFNNGLCKKGPKNTYMCECKQGFAGERC**
ERNAGKKLEA*

B

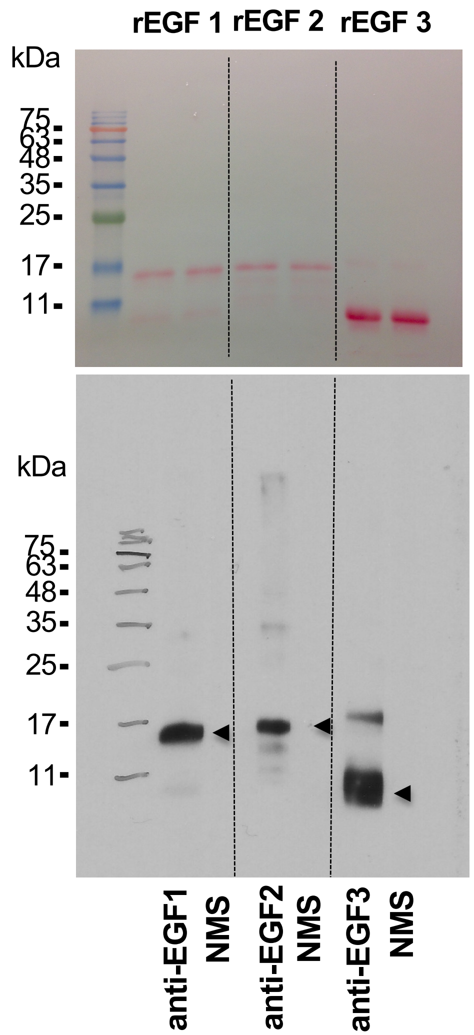

Fig. S1. Expression of recombinant EGF domains. A. Three protein sequences encompassing EGF domains (highlighted in red). The Is86 EGF-1 domain is derived from *Is86-1*, EGF-2 from *Is86-2*, and EGF-3 from common sequences of both *Is86-1* and *Is86-2*. **B.** Is86 EGF domain expression and antiserum generation. Three recombinant EGF-1, -2, and -3 proteins were stained with Ponceau (upper panel) and immunoblotted using either the specific EGF domain antiserum (EGF-1, -2, or -3), or normal mouse serum (NMS). The specific reactions are indicated with arrow heads.

Fig. S2

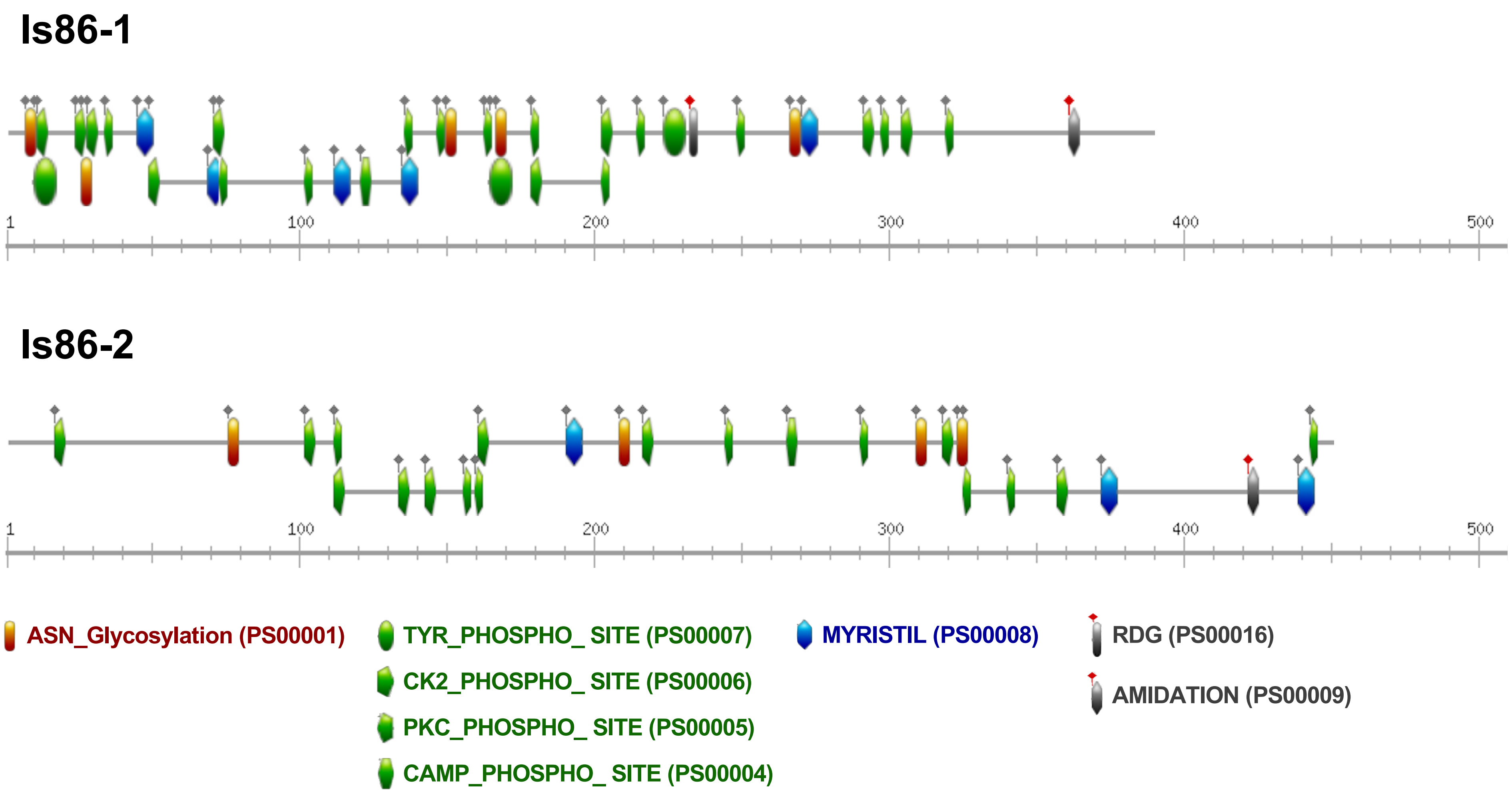

Fig. S2. Patterns and posttranslational modifications on Is86 homologs. *In silico* search using PROSITE database (https://prosite.expasy.org/index.html).

Fig. S3

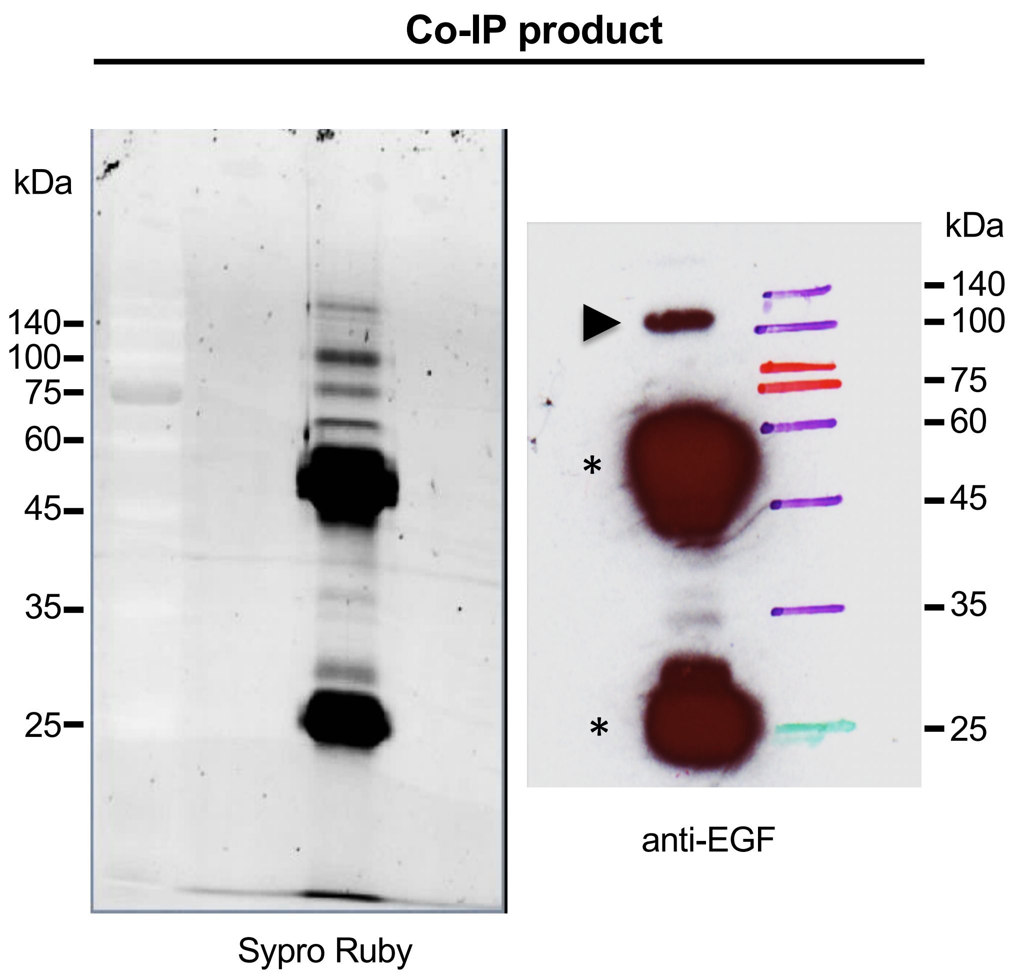

Fig. S3. Immunoprecipitation of native Is86 protein using anti-EGF antibodies. Pooled anti-EGF serum was used to immunoprecipitate the native Is86 protein from unfed adult tick gut extract. The precipitated products were separated on SDS-PAGE gel, stained with Sypro Ruby (left panel), and immunoblotted using pooled antiserum against EGFs (right panel). The native Is86 protein that specifically reacted around 100kDa is indicated with an arrowhead. These two major bands are likely to be the IgG heavy chain and light chain, derived from IP using protein G beads.

Fig. S4

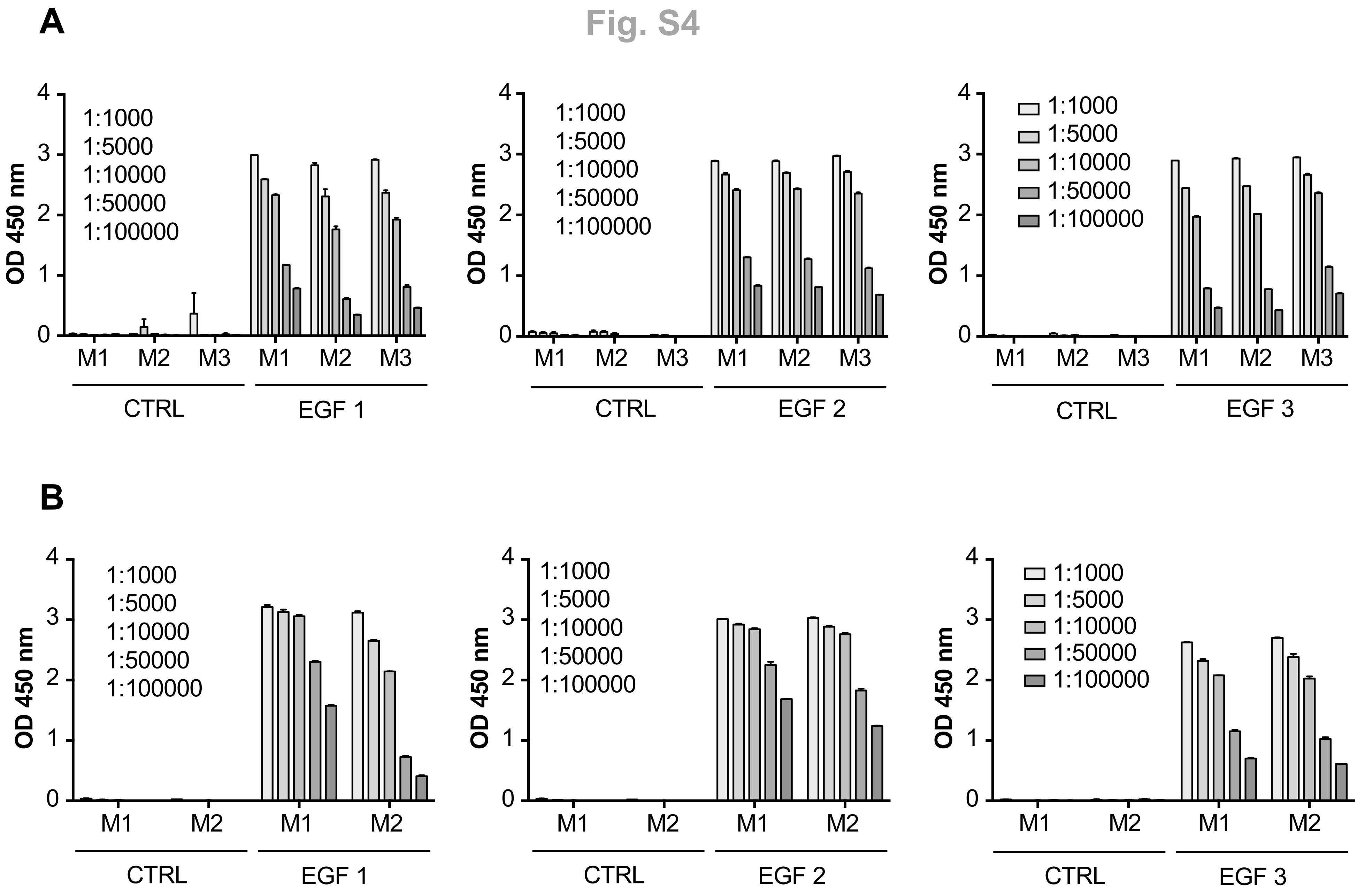

Fig. S4. Antibody titers in mice immunized with recombinant EGF domains. ELISA showing the development of high-titer antibodies in mice immunized with all recombinant EGFs and used in *B. burgdorferi* transmission **(A)** and acquisition **(B)** experiments. The wells were coated with rEGFs in duplicates and probed with either the corresponding EGF domain antiserum (1:1000 to 1:100000) or control (adjuvant only) serum.

Fig. S5

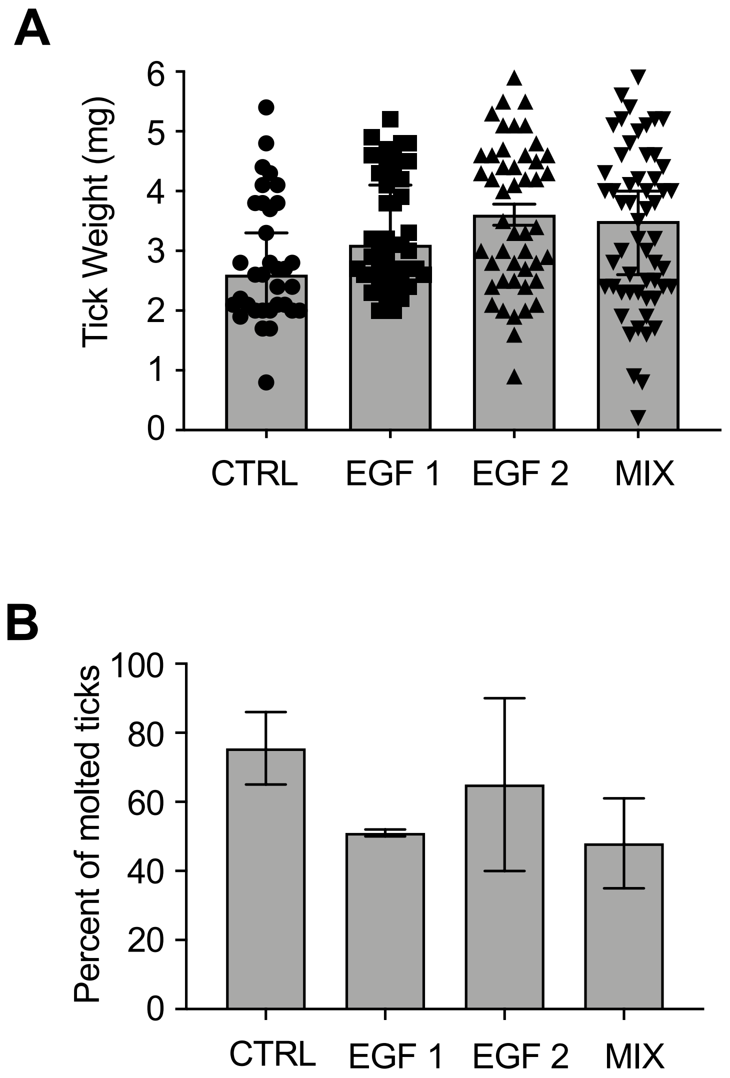

Fig. S5. **Immunization with recombinant EGF domains does not affect major feeding parameters of naïve *I. scapularis* nymphal ticks. A.** Scatter chart with white bars showing the median weight of individually plotted fully replete ticks (n=34, 47, 47, 57/group, respectively) collected after tick feeding, with error bars showing confidence intervals. **B.** Bar chart indicating the mean percentage of adult ticks molted from nymphs two months after feeding to repletion (error bars with SEM are indicated).

Fig. S6

Full-length blots as presented in Fig. 2C

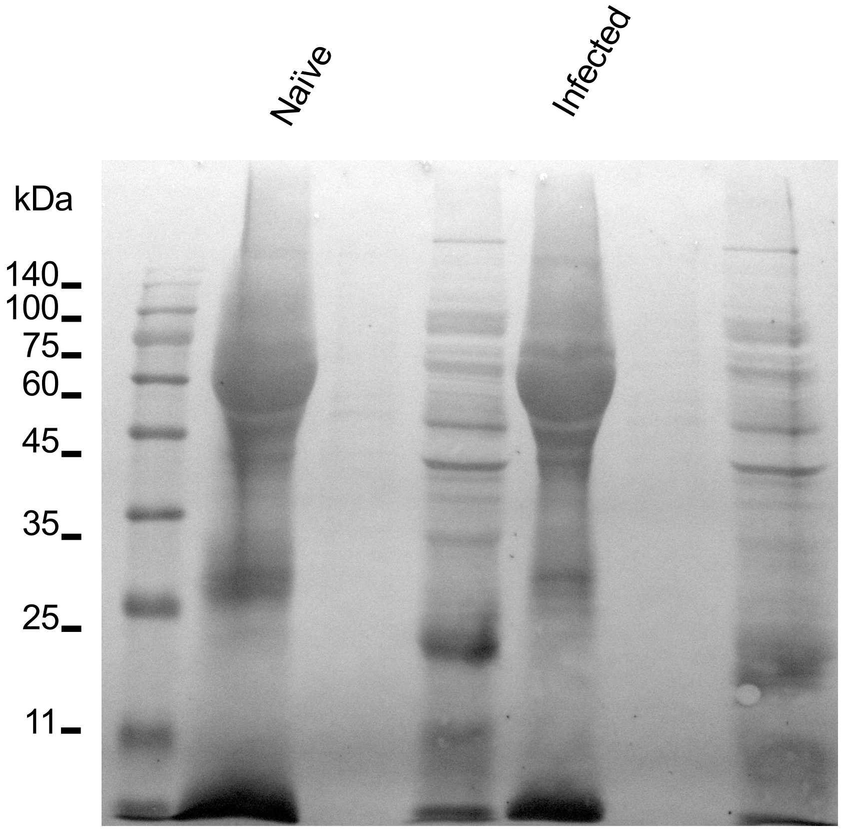

Ponceau S

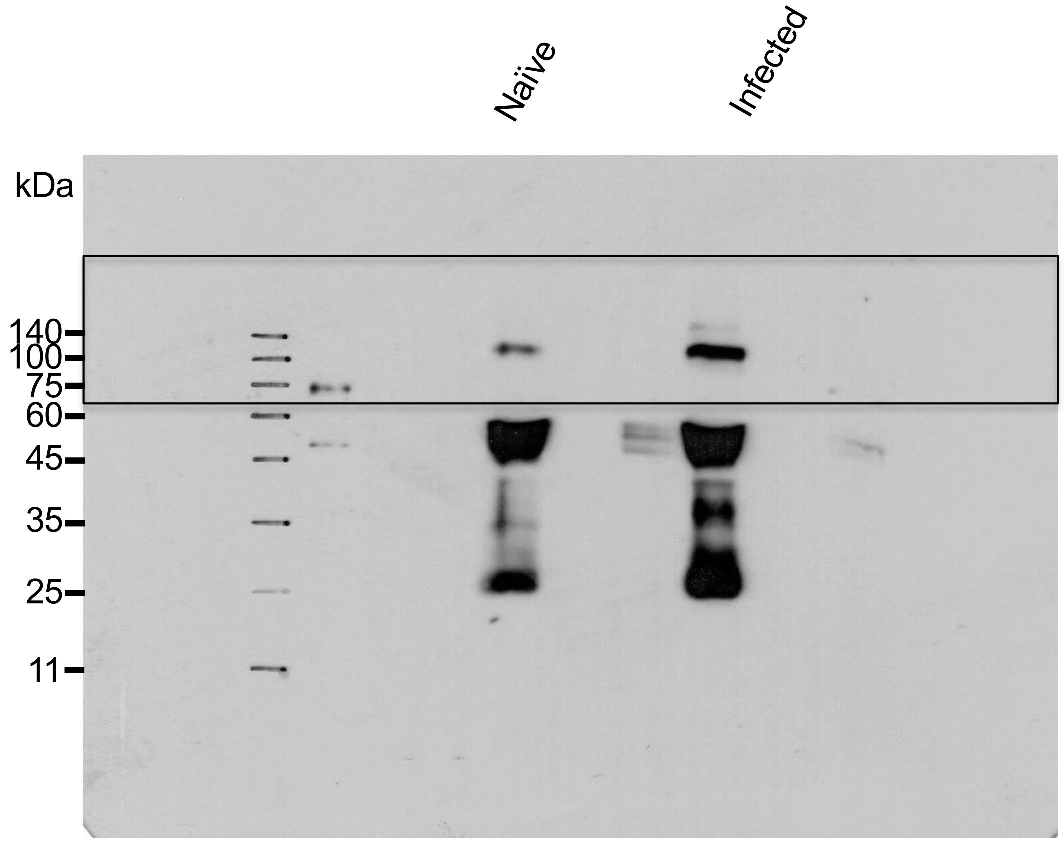

Anti-EGF

Fig. S6. Full-length blots as presented in Fig. 2C. For description, please refer to the Fig. 2 legend.

Fig. S7

Full-length Immunoblots as presented in Fig. 3

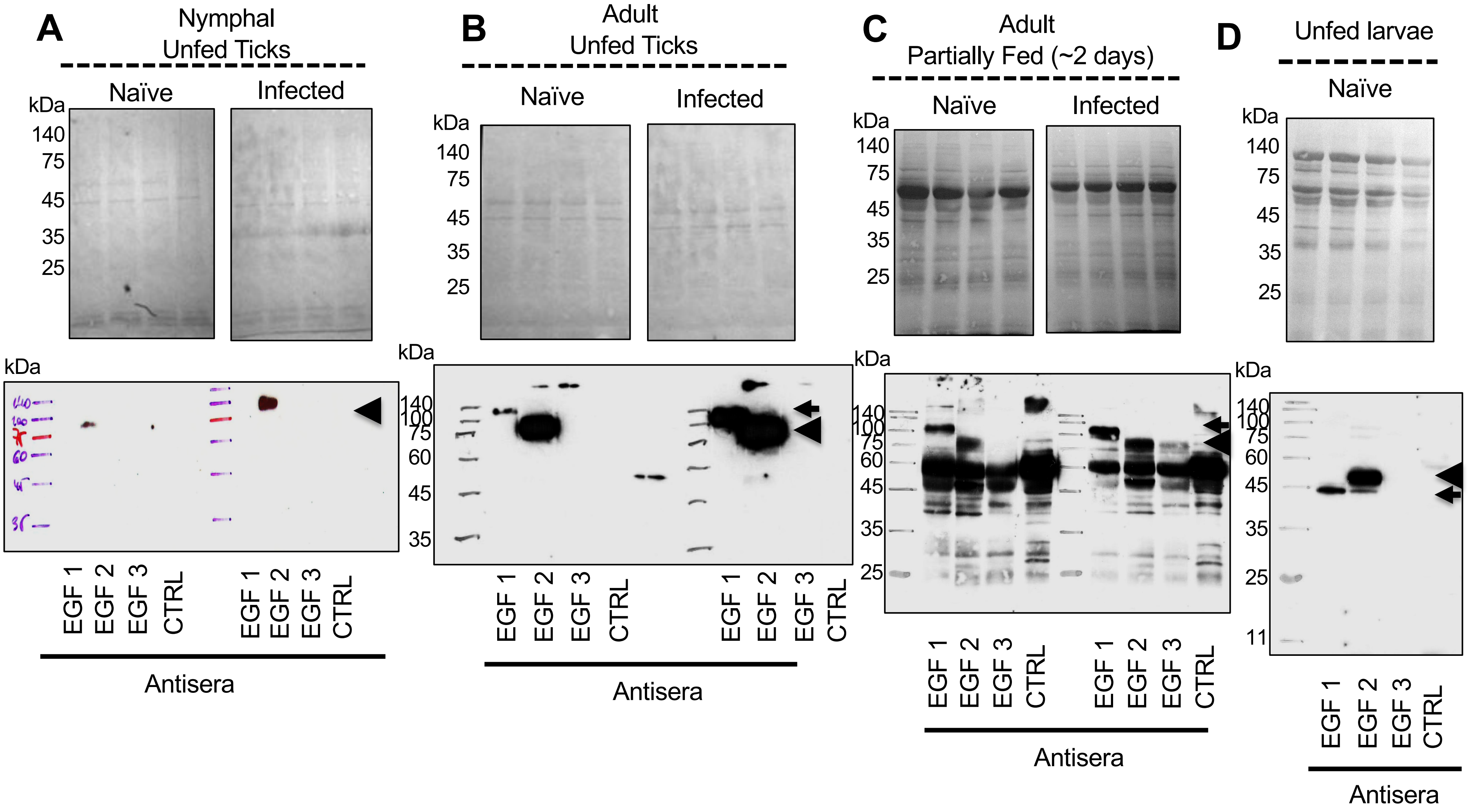

Fig. S7. Full-length immunoblots as presented in Fig. 3. For description, please refer to the Fig. 3 legend.

Table S1. Oligonucleotide primers used in the current study

Primer name	5'-sequence-3'	Purpose
Is86-1_For	ATGAAGGACCTATACGAAAACTGC	Full length ORF TA cloning
Is86-1_Rev	TTAGGCGAAACCAAACAACCAC	Full length ORF TA cloning
Is86-2_EXT_For	GTCATGTGTTCGCCACAAAC	Nested PCR for full length ORF TA cloning
Is86-2_EXT_Rev	TCGGACTTCAATGACCAACA	Nested PCR for full length ORF TA cloning
Is86-2_INT_For	ATGAAGGACCTATACGAAAACTG	Nested PCR for full length ORF TA cloning
Is86-2_INT_Rev	TTAGGCCAAACCAAACATCCAC	Nested PCR for full length ORF TA cloning
Is86-1_EGF1_For	ggtggtcatatgatgTGCACGGCAAAAGTC	EGF subcloning
Is86-1_EGF1_Rev	ggtggtctcgagtcaGCACGTGATATTTCTGG	EGF subcloning
Is86-2_EGF2_For	ggtggtcatatgatgTGTACGGCAAACGTAACT	EGF subcloning
Is86-2_EGF2_Rev	ggtggtctcgagtcaGCACATTTGATTGCC	EGF subcloning
Is86-1+2_EGF3_For	ggtggtcatatgatgACAACCACGTTGAACAA	EGF subcloning
Is86-1+2_EGF3_Rev	ggtggtctcgagtcaCGCCTCAAGCTTTTTAC	EGF subcloning
Is86-1_dsRNA1_For	taatacgactcactatagggCGCTACTGCAAACCTGGATA	RNAi
Is86-1_ dsRNA1_Rev	taatacgactcactatagggAGTCCGCACGTGATATTTCT	RNAi
Is86-1_ dsRNA2_For	taatacgactcactatagggACGTCACCTTACAGCGATGA	RNAi
Is86-1_ dsRNA2_Rev	taatacgactcactatagggTTAATCGTCCCGATGTAACC	RNAi
Is86-2_dsRNA1_For	taatacgactcactatagggCGGAAGAGTTTACTGCGATT	RNAi
Is86-2_ dsRNA1_Rev	taatacgactcactatagggCGCACTTCGTGGTACACTTA	RNAi
Is86-2_ dsRNA2_For	taatacgactcactatagggGGTGCACTTGCATCTATCCT	RNAi
Is86-2_ dsRNA2_Rev	taatacgactcactatagggGCCCTGATTTTACTTCACCA	RNAi
Is86_qPCR_ For	GCATAAGCATTCCTTGCTTC	qPCR
Is86_qPCR_ Rev	CTTGTTTGCATTCGCACATA	qPCR
flaB_For	GAAATTGCCAACAGTAGTCC	qPCR
flaB_Rev	GGTCTTCTTCTTTTGGGTTT	qPCR
Tick β-actin_For	AGAGGGAAATCGTGCGTGAC	qPCR
Tick β-actin_Rev	CAATAGTGATGACCTGGCCGT	qPCR
Tick rps4_For	GGTGAAGAAGATTGTCAAGCAGAG	qPCR
Tick rps4_Rev	TGAAGCCAGCAGGGTAGTTTG	qPCR
